# Desert ants do not acquire and use a three-dimensional global vector

**DOI:** 10.1186/1742-9994-4-12

**Published:** 2007-05-03

**Authors:** Gunnar Grah, Rüdiger Wehner, Bernhard Ronacher

**Affiliations:** 1 Department of Biology, Humboldt-Universität zu Berlin, Invalidenstrasse 43, D-10099 Berlin, Germany; 2 Institute of Zoology/Neurobiology, University of Zurich, Winterthurerstrasse 190, CH-8057 Zurich, Switzerland

## Abstract

**Background:**

Desert ants (*Cataglyphis fortis*) are central place foragers that navigate by means of path integration. This mechanism remains accurate even on three-dimensional itineraries. In this study, we tested three hypotheses concerning the underlying principles of *Cataglyphis*' orientation in 3-D: (1) Do the ants employ a strictly two-dimensional representation of their itineraries, (2) do they link additional information about ascents and descents to their 2-D home vector, or (3) do they use true 3-D vector navigation?

**Results:**

We trained ants to walk routes within channels that included ascents and descents. In choice tests, ants walked on ramps more frequently and at greater lengths if their preceding journey also included vertical components. However, the sequence of ascents and descents, as well as their distance from nest and feeder, were not retraced. Importantly, the animals did not compensate for an enforced vertical deviation from the home vector.

**Conclusion:**

We conclude that *Cataglyphis fortis *essentially represents its environment in a simplified, two-dimensional fashion, with information about vertical path segments being learnt, but independently from their congruence with the actual three-dimensional configuration of the environment. Our findings render the existence of a path integration mechanism that is functional in all three dimensions highly unlikely.

## Background

Desert ants (*Cataglyphis fortis*) are scavengers, inhabiting the barren salt-pans of the North African Maghreb. In search for prey, these ants traverse their habitat on tortuous paths. In the absence of landmarks that could guide them [1], they head on a direct way towards their nest as soon as they have encountered a suitable booty [2,3]. The orientation mechanism that enables *Cataglyphis *to accurately return to their – mostly inconspicuous – nest entrance is path integration [4], i.e. the ability to combine the individual segments of an outbound journey in order to create a global home vector, which points back to the nest with the correct distance and compass direction.

One prerequisite for path integration is a reference system for the compass direction of a walked course. *Cataglyphis *utilizes both the position of the sun [5,6] and, in particular, the pattern of polarized skylight [7-10]. The other requirement of a path integrator is some form of odometer, which is probably implemented in desert ants as a kind of step counter [11,12], whereas self-induced optic flow and energy expenditure have been ruled out as the predominant sources of information [13-16].

Remarkably, the ants' path integrator still functions accurately when parts of the journey lead over a series of hills, thereby increasing the actual walking distance compared to the bee-line distance that is relevant for determining the home vector [16,17]. A three-dimensional outward journey, followed by a homebound trip carried out on level ground, causes no directional error [18]. This leads to the conclusion that the ants' path integrator correctly incorporates distances that were walked on slopes with their corresponding *ground distance*, not the actual *walking distance*. This ability to re-calculate a path length to its ground distance enables desert ants to orientate accurately in undulating terrain even if the path towards a food source and the way back to the nest lead through areas of different topography.

Based on these observations we now ask how sophisticated a desert ant's representation of its three-dimensional environment really is. As a starting point, we formulated three hypotheses that assume mechanisms of increasing complexity. These hypotheses lead to different predictions of how the ants would respond in test situations after specific forms of training (see Results): 

**Hypothesis A: **The ants' path integration module works essentially in the horizontal plane; the ants store no information about the 3-D component of their environments. For this hypothesis we assume that distances walked on slopes are corrected to ground distance in real-time, but any additional information about the three-dimensionality of an itinerary is discarded. The world as perceived by the ant is a plane projection without vertical expansion. 

**Hypothesis B:** The ants combine their 2-D path integration as outlined above with additional information about the three-dimensional structure of a walked path. E.g., motor commands for a vertical change of direction could be coupled to the current status of the home vector, or to specific landmarks. The ants could be prompted to execute these motor commands either in the sequence that was learnt on preceding foraging forays, or at specific distances from the end points of their trip. Thus, information about vertical changes of direction would be remembered in a form of procedural knowledge, which could already be demonstrated for 2-D itineraries [19]. If such changes fail to appear along a known route, this could affect the ants' homing behavior. This hypothesis is similar to the described orientation by local vectors that *Cataglyphis *uses in the horizontal plane. Here, the animals link local vectors [20] and motor commands [20,21] to familiar landmarks, which ultimately connect in sequence to complete routes defined by local vectors (shown in *Melophorus bagoti*: [22]).  

**Hypothesis C:** The ants possess a path integration module that is able to compute a correct vector with respect to all three dimensions, i.e. a true 3-D vector. In principle, a 2-D representation of itineraries that corrects walking distances on slopes to their respective ground distance would be sufficient to navigate accurately between the nest and a food source. However, the desert ants' path integrator is prone to systematic errors [4], and locates its goal with an uncertainty that increases with the distance walked [23,24]. Under these circumstances, information could be valuable whether a food source is located at the top or the bottom of a slope. Sandy deserts are also an example of undulating terrain that is lacking unambiguous visual landmarks, which otherwise could help to home in on a known food source [25]. If the vertical component of such a 3-D vector cannot be followed, e.g. due to the lack of descent/ascent opportunities, this should affect the ants' homing behavior as well.

Unfortunately, it is difficult to discriminate experimentally between these hypotheses. In order to unequivocally prove, for example, that ants do compute a true 3-D vector, it would be necessary to perform the analogous experiments as in 2-D [4,26]. However, it is not feasible to offer an ant the same freedom to approach and to choose any points in 3-D space as it is possible in 2-D. For this reason, we had to reduce the complexity of the 3-D problem to experimentally manageable pieces. Although none of these tests by themselves allows for an unambiguous decision in favor of any of the outlined hypotheses, their combination allows for rather firm conclusions about the level of sophistication of *Cataglyphis*' orientation in 3-D space.

## Results

### Experiment 1 and 2

**Figure 1:**
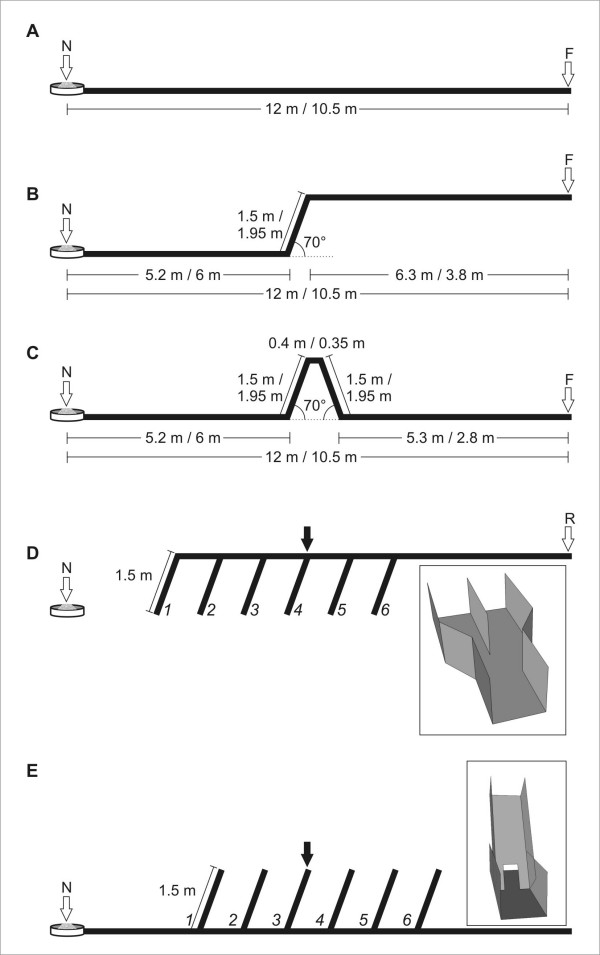
**Schematic lateral view of channels used in training and test situations**. Ants were trained from the nest ("N") to visit a feeder ("F") at the end of a series of channels. (A) depicts the situation for flat training. (B) shows the situation for ramp training, leading to an elevated feeder. (C) illustrates the set-up for Λ training, which led the ants to a feeder at ground level over an artificial "hill". Dimensions refer to experiment 1 and experiment 2, separated by "/". (D) In experiment 1, homebound ants were placed individually in a test channel at the release point ("R"). At six points with distances of 1.6 m between them, ants could choose either to continue walking horizontally, or climb down on a ramp. The "decision point" consisted of a widening in the channel (D, inset), where one side would continue horizontally, and the other one lead downwards. (E) In experiment 2, ants leaving the nest ("N") were led individually into a test channel that offered six ascending ramps, each at 1.5 m distance from the next. Gateways at the ramp bases (E, inset) allowed animals to climb up or pass through and continue walking horizontally. In D and E, the position of the ramp marked by a black arrow corresponds to that of the upward training ramp.

Different groups of ants were trained in one of three different training paradigms (Figure 1A–C). These consisted of walking within a channel system either to a feeder at ground level (flat training; Figure 1A), or to an elevated food source ("ramp training", Figure 1B), or to pass a hill while being trained to a feeder at ground level (Λ training, Figure 1C). This last training was supposed to result in the same global vector between feeder and nest as in the flat training, but to include a vertical component in the path, as in the ramp training.

According to hypothesis A (the ant's representation is restricted completely to 2-D), one would expect that when tested on slopes, ants trained to an elevated feeder would behave similarly as ants that were trained on flat ground. If ants would compute a true 3-D vector (hypothesis C), a first expectation would be that they behave similarly after flat and Λ training, as in both cases the global vector pointing to the feeder or to the nest has no vertical component. If ants work with an "operational" rule (hypothesis B), e.g. to search for an ascent/descent after having covered a certain ground distance, the expectation is that – if offered several choices – they will prefer an ascent/descent located at the training distance. Further expectations will be mentioned below, together with the respective experiments.

### Experiment 1: Homebound tests

**Figure 2:**
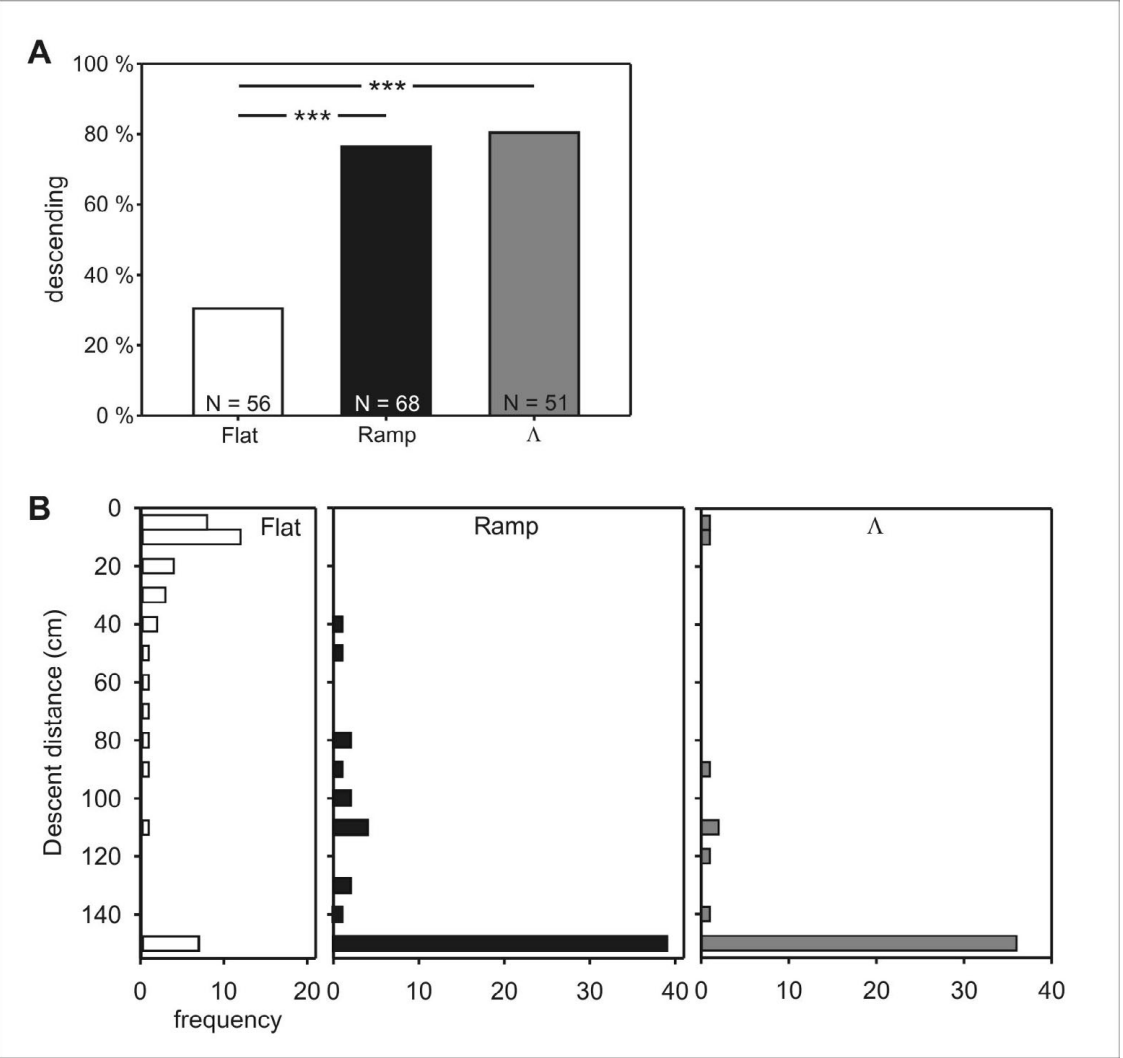
**Experiment 1: Proportion of descents and descent distances after flat, ramp, and Λ training**. (A) The criterion defining a descent was for ants to walk more than 20 cm without turning around on one of the offered ramps. The frequencies of ants that chose to descend differed between the three training paradigms (*p *< 0.001, χ^2 ^homogeneity test). The pair-wise comparison was carried out with Fisher's exact test and a Bonferroni correction for multiple comparisons. (B) Frequency histograms of distances that ants walked on descending ramps before turning around for the first time after flat training, ramp training, and Λ training. Ants that made a full descent without ever turning around are plotted in the bottom bar. Results after ramp and Λ training did not differ among each other, but differed strongly from those after flat training (*p *< 0.001, Kruskal-Wallis H-test; Games-Howell post-hoc test for pair-wise comparisons).

When beginning their way from the feeder back to the colony, food-carrying ants were individually placed or guided into a system of elevated channels that offered six "decision points". At each of them, the animal could either continue walking horizontally, on the elevated level, or descend a ramp back to the ground (Figure 1D). The proportion of ants that climbed down the ramps for more than 20 cm without turning around was much smaller after flat training than after ramp or Λ training, while the percentages did not differ for the latter two training paradigms (Figure 2A). The training scheme had an even more pronounced influence on the distance walked on the ramps (Figure 2B). Most of the flat trained animals, if they attempted a descent at all, turned around after only a short distance and walked upwards again (median descent = 20 cm; Figure 2B). These early first turns stand in stark contrast to the behavior of ramp trained and Λ trained ants, the majority of which descended the ramp to its full length of 150 cm (both medians = 150 cm; Figure 2B). The results of flat trained ants differed strongly from those of ramp and Λ training, which did not differ among each other.

**Figure 3:**
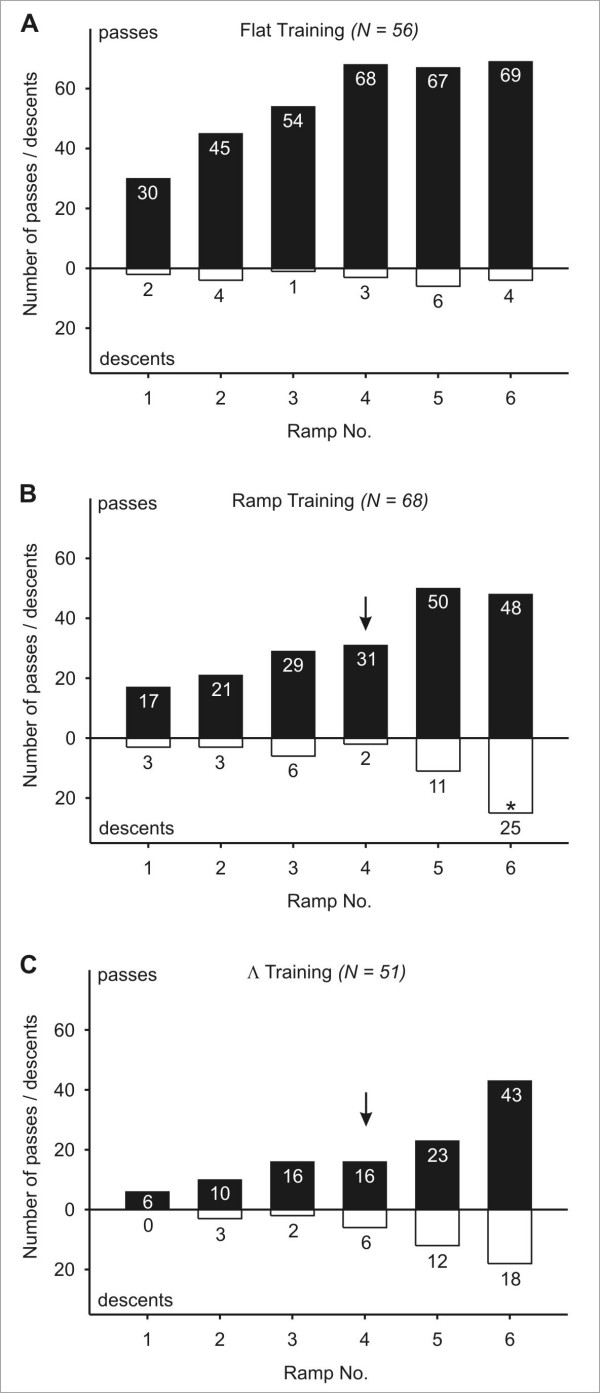
**Experiment 1: Choice between different ramps**. Decisions of ants to descend (white bars) or to pass (black bars) at the six ramps that were on offer. Ramp No. 1 was located nearest to the nest; ramp No. 6 was nearest to the position of the feeder and was thus encountered first on the examined homeward runs. The black arrow indicates the ramp that corresponded to the ascending ramp during training. (A) Flat training; (B) Ramp training: a significantly higher proportion of animals descended at ramp No. 6 (*p *< 0.05, χ^2 ^homogeneity test and squared standardized residuals); (C) Λ training. Flat and Λ training: Due to low expected frequencies, no deviation from a uniform distribution could be detected.

The numbers of passes and descents that ants made during their homebound runs are classified according to the ramp positions in Figure 3. Only the ramp training resulted in a non uniform distribution of descents between the ramps on offer, caused by an extraordinarily high number of descents at ramp No. 6, the first decision point for a returning ant (Figure 3B). In the case of flat and Λ training (Figure 3A,C), the overall numbers of full descents were too small to identify heterogeneous distributions of the ants' choice between ramps. As can be seen by the length of the bars in Figure 3, the number of total decisions made at the ramps decreased markedly from ramp 6, which was the first one encountered on the homebound trip, towards ramp 1. This was particularly true for the ramp and Λ training situations, where a high proportion of ants descended the full length of the first ramp that they encountered, thus ending their respective test runs.

If we applied a softer criterion by comprising all descents that went further than 20 cm in the "descent" category, this did not change the outcome considerably (Additional file 1). Please note that the total numbers of decisions for each ramp do not correspond exactly between the two criteria. The underlying reason is that under the two criteria the analysis of an ant's run ended at different points. Consider for example an ant that chooses on its first descent to climb down a ramp for 50 cm. According to the "hard" criterion (depicted in Figure 3), this incomplete descent was considered as a decision against this respective ramp, counted consequently as a pass, and the ant's path would be analyzed further. Under the "soft" criterion however (documented in Additional file 1), a descent of 50 cm (i.e., of more than 20 cm) was seen as a decision in favor of this ramp, counted as a descent, and the run was considered to be terminated.

After flat training, ramp choice was distributed homogenously (*p *> 0.1, χ^2 ^homogeneity test and squared standardized residuals). Ramp training resulted in a heterogeneous distribution (*p *< 0.01), with a disproportional number of ants descending at ramp No. 6. To a lesser extent, the relatively small number of ants descending at ramp No. 4 also contributed to the detected heterogeneity. The number of descents recorded after Λ training was still not sufficient to detect heterogeneity.

#### Tests in a flat channel

If the ants operate exclusively in 2-D (hypothesis A), they should always search for their nest at the correct ground distance when released for their homebound run in a flat test channel – irrespective of the training paradigm. If, on the other hand, *Cataglyphis *couples the memory of an ascent or descent with certain values of her home vector, one could expect an increased search density at a distance corresponding to the position of the ramp during training. This would be akin to the "procedural knowledge" that could be demonstrated in 2-D experiments [19]. In order to check these predictions, we trained ants to a feeder, following the same three paradigms as described above (flat, ramp, Λ). Ants were then taken from the feeder and placed into a flat channel, and the first U-turns of their homebound runs were recorded.

**Figure 4:**
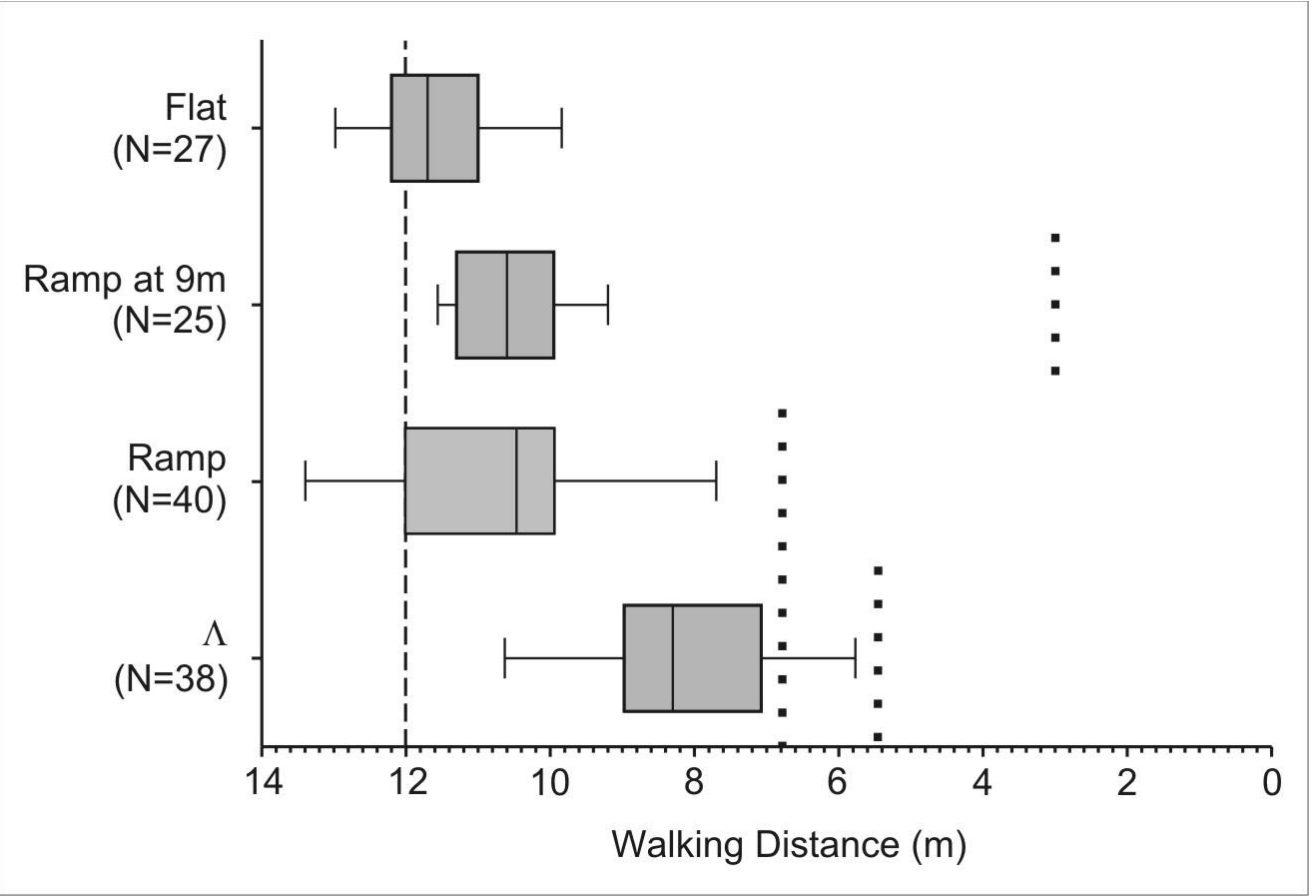
**Experiment 1: Distribution of first U-turns in a flat channel on a homebound run**. The point of release was at 0 m. Dashed line: expected position of the nest. Dotted lines: positions of ramps in the preceding training situations. The distribution of U-turns differed between Λ and all other training paradigms (*p *< 0.001), and between flat training and training with ramp position at 9 m from the nest (*p *< 0.05; Kruskal-Wallis H-test and Games-Howell post-hoc test for pair-wise comparisons).

The distances at which ants begun their search differed between the training paradigms "flat", "ramp", and "Λ" (Figure 4). Flat training (median of first U-turns: 11.7 m) resulted in search distances that came closest to the relative position of the nest, which corresponded to the 12 m mark in the test channel. All other training situations resulted in animals displaying shortened homing distances. This "undershooting" was most pronounced after Λ training (median of first U-turns: 8.3 m). After ramp training, the animals also carried out a slightly truncated search for the nest (median of first U-turn: 10.45 m), although a larger scatter leads to non-significant differences to the results from flat training. In order to unequivocally decide whether the ants searched for the nest or for the beginning of the descent, we included an additional training, in which the ramp was located at 9 m distance from the nest. Under this condition, an ant had experienced on its homebound run the beginning of the ramp already 2.5 m after the feeder. As Figure 4 shows, the centers of search (median for "ramp at 9 m": 10.6 m) did not differ between the two forms of ramp training, i.e. they were not influenced by the position of the ramp.

### Experiment 2: Outbound tests

**Figure 5:**
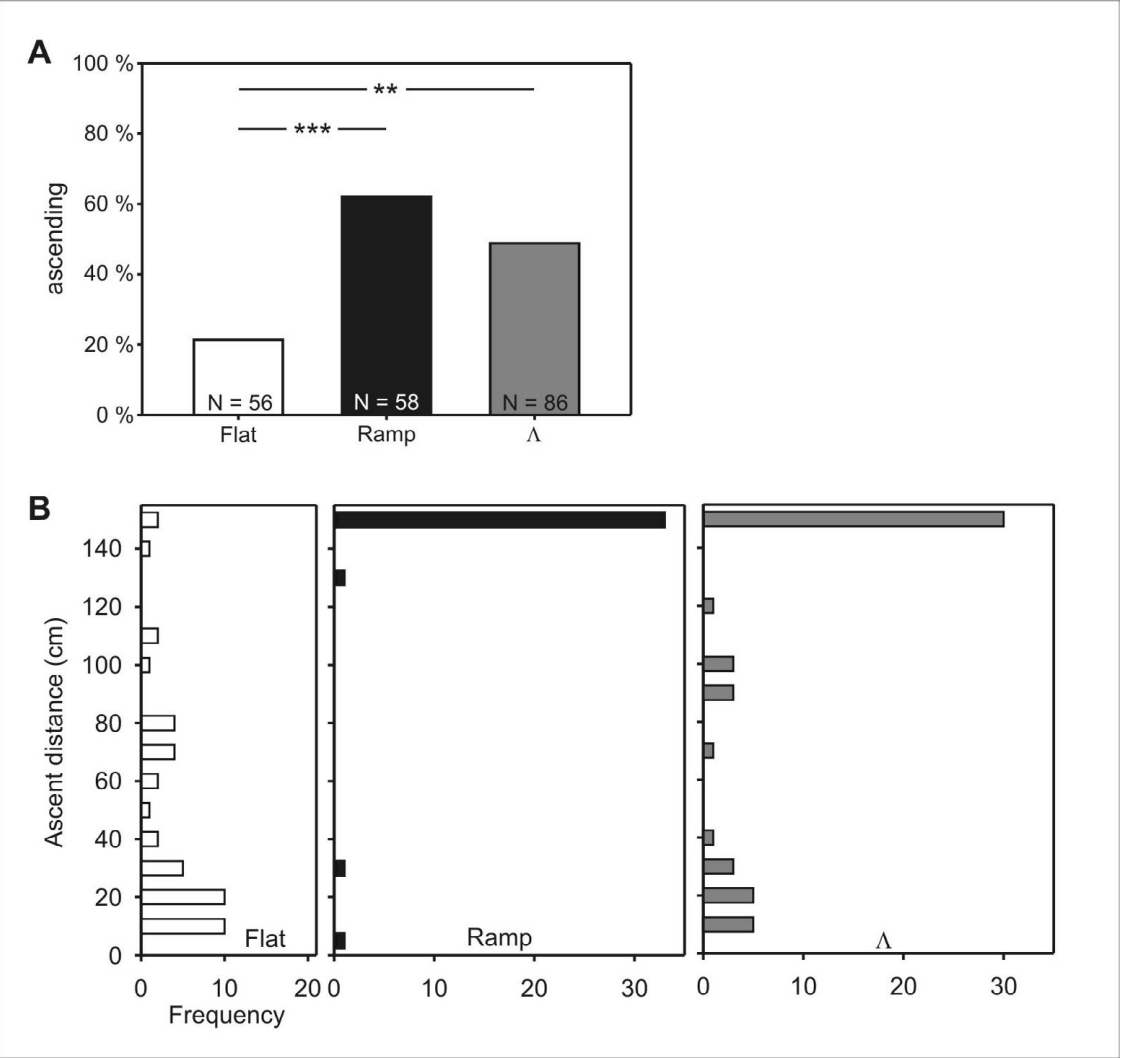
**Experiment 2: Proportion of ascents and ascent distances after flat, ramp, and Λ training**. (A) The criterion defining an ascent was for ants to walk more than 20 cm without turning around on one of the offered ramps. The frequencies of ants that decided to ascend differed between the three training paradigms (*p *< 0.001, χ^2 ^homogeneity test). The pair-wise comparison was carried out with Fisher's exact test and a Bonferroni correction for multiple comparisons. (B) Frequency histograms of distances that ants walked on ascending ramps before turning around for the first time after flat training, ramp training, and Λ training. Ants that made a full ascent without ever turning around are plotted in the top bar. Ascent heights differed strongly between all three training paradigms (*p *< 0.001, Kruskal-Wallis H-test; Games-Howell post-hoc test for pair-wise comparisons).

After training in almost identical fashion to the Homebound test (see the Materials and Methods section), we tested ants that were on their outward trip from the nest to a feeder that they had frequently visited before. Individual ants were given access to a test channel by means of a switch near the nest entrance. The animals had six ramps at which they could choose either to ascend or to pass through a central gateway (Figure 1E, inset). As in the Homebound test, the type of training that the ants underwent prior to testing had a marked influence on the number of individuals that stepped onto a ramp and continued to climb on it for more than 20 cm (Figure 5A). Fewer individuals chose to climb up after flat training than after ramp or Λ training.

The distribution of distances that ascending ants walked on the ramps before turning around for the first time mirrors the results of the Homebound tests (compare Figure 2B with Figure 5B). After flat training, most of the ants' U-turns were located in the lower half of the ramps (Figure 5B; median = 30 cm). In contrast, almost all of the ascending ants from ramp training and the majority of animals that underwent Λ training, climbed the full length of the ramps (Figure 5B; both medians = 150 cm). A statistical comparison of the ascent distances confirmed strong differences between all three training paradigms.

**Figure 6:**
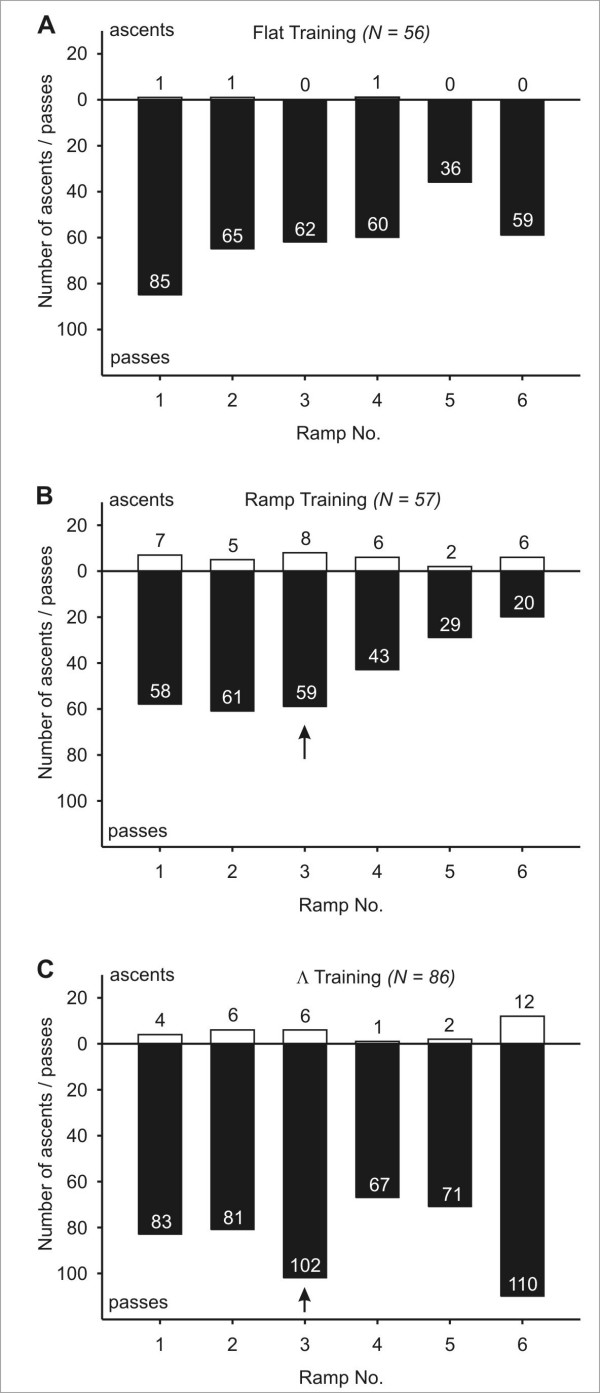
**Experiment 2: Choice between different ramps**. Decisions of ants to ascend (white bars) or to pass (black bars) at the six ramps that were on offer. Ramp No. 1 was located nearest to the nest, and was thus encountered first on the examined outbound runs. The black arrow in (B) and (C) indicates the ramp that corresponded to the ascending ramp during training. (A) Flat training; (B) Ramp training: the high number of ascents at ramp No. 6 did not result in a non uniform distribution (*p *> 0.1, χ^2 ^homogeneity test); (C) Λ training. Flat and Λ training: Due to low expected frequencies, a non uniform distribution could not be detected.

The distribution of ascents over the six ramp positions is shown in Figure 6. In the case of flat training (Figure 6A), only 3 out of 370 total decisions of ants at the different test ramps resulted in a complete ascent, prohibiting any conclusions about their distribution.

In spite of the high relative value of ascents at ramp No. 6 (the ramp furthest away from the nest), where 6 out of 26 decisions resulted in climbing up, ramp training did not cause a heterogeneous distribution in the number of ascents compared to passes (Figure 6B). In the case of Λ trained ants, we could also observe a trend to prefer ramp No. 6 (Figure 6C). However, the overall number of ascents was also too small to confirm a heterogeneous distribution. Here too, we applied a softer criterion for defining ascents, in order to rule out that our strict definition of ascents masked any effects of the different training situations, and considered all ascents that continued for more than 20 cm (Additional file 2). Now, the data retrieved from all training paradigms was sufficient to carry out a statistical analysis of heterogeneity. In the case of flat and ramp training, no preference or rejection of any ramp could be detected (*p *> 0.05, χ^2 ^homogeneity test). In Λ training however, the trend observed under the strict criterion could be confirmed, with an over proportional number of ants climbing up on ramp No. 6 (*p *< 0.001, χ^2 ^homogeneity test and squared standardized residuals).

### Experiment 3: Induction of a negative vertical vector component

**Figure 7:**
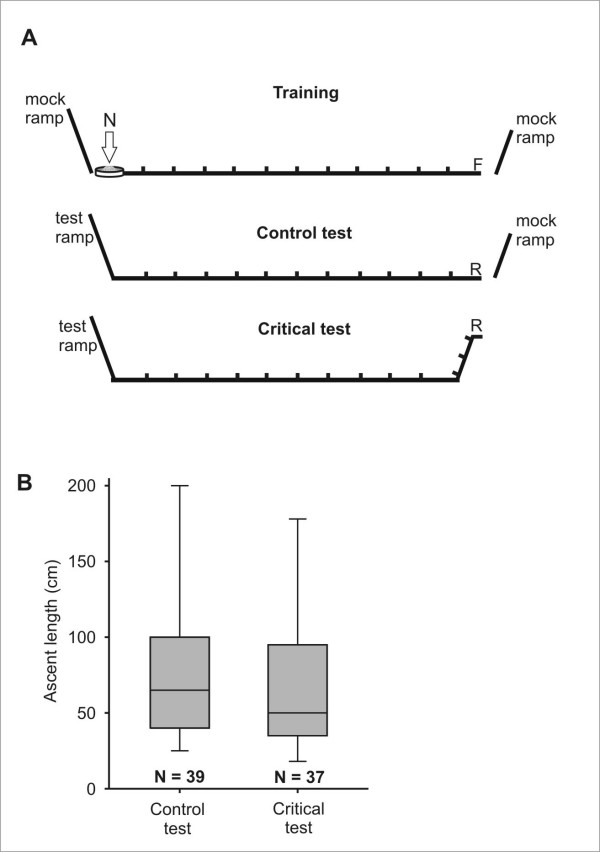
**Experiment 3: Induction of a negative vertical vector component**. (A) Schematic lateral view of the channels used in training, control, and critical test. Small marks indicate the presence of landmarks within the channel (refer to text). Mock ramps in the training and control situation served to provide similar visual environments in all three situations. (B) Distribution of lengths of the first ascent in the control and critical test. The results from both test situations did not differ from each other (p > 0.1, Mann-Whitney U-test).

If the ants do acquire a true 3-D vector (hypothesis C), it should be possible to specifically and separately influence the vertical component of the 3-D vector. The rationale of this experiment was to train ants essentially in a *flat channel *to a feeder on level ground (Figure 7A, "Training"). After several visits at the feeder, ants were transferred for the critical experiment to the elevated end of the test channel (Figure 7A, "Critical test"; "R" marks the release point), so that on their homebound run they first experienced a descent. At the nest position, the test channel ended in an ascending ramp (2 m long), and the length of ascents on it was recorded. If during the "enforced" descent within the test channel the ants had built up a negative vertical vector, they should be eager to ascend again on the test ramp. As a control, a different group of ants was released on level ground within the test channel at the same distance from the nest as in the critical test (Figure 7A, "Control test"; "R" marks the release point). Note that the training was always completely flat. Hence, according to the results gained from flat-trained animals in experiments 1 and 2, the expectation was that ants in the control should not ascend very far on the ramp located at the nest position. In order to facilitate the descents in the critical test, in this experiment both the training and the test channels were lined with landmarks that were inconspicuous on the outbound runs, but conspicuous on the homebound runs (see Methods section).

The results are very clear (Figure 7B): As expected, ants that were trained and tested in the flat channels did not ascend very high on the test ramp (median: 65 cm). Note that in this test channel the ants had no opportunity to continue their path in the horizontal plane (as in experiment 1 or 2), but were forced to continue on the ascending ramp. This probably explains the larger ascent height, as compared to Figure 5B. Most importantly, however, the median ascent height did not differ between the critical test (with forced descent, N = 37) and the control (without descent, N = 39). This shows clearly that the ants did not accumulate a negative vertical vector component during this "enforced" descent, which would have prompted them to compensate for this vertical vector proportion on the ascending ramp. This experiment renders a vertical vector component, and thus a true 3-D vector, very unlikely.

## Discussion

In this paper, we aim to determine which properties of a three-dimensional path are stored and available to recall in desert ants, *Cataglyphis fortis*, by comparing their behavior in three different training paradigms. The most conspicuous result is the close similarity of the ants' behavior after ramp and Λ training, while the flat training resulted in a different behavior. These differences enable us to identify some features of the ants' orientation mechanism when confronted with a three-dimensional itinerary. Furthermore, the results from experiment 3, which specifically tested the ants' orientation pertaining to a vertical vector component, do not provide supportive evidence for a 3-D path integrator.

### Desert ants do not completely discard information about descents and ascents

Earlier experiments had shown that *Cataglyphis *ants are able to compute ground distances when walking on slopes [16,17] and to correctly incorporate this information into their path integration module [18]. A first simple hypothesis, which is fully compatible with these earlier results, would be that the ant's path integrator operates exclusively in 2-D, and that the animals have no further access to information about the 3-D structure of their environment after having corrected the walking distance to ground distance. The expectation under this hypothesis is that upon encountering slopes, ants should show similar behavior after flat and ramp training. The data, however, are clearly not consistent with this expectation. The proportion of ants that descended or ascended on one of the test ramps in Homebound and Outbound tests, respectively, differed strongly between flat trained and ramp trained animals (Figure 2A and 5A).

When considering only those animals that chose to descend or ascend in Homebound and Outbound tests, the distance that was walked on ramps provides additional, independent evidence for the different effects of the training paradigms (Figures 2B and 5B): Not only did fewer ants step onto ramps after flat training, but those flat trained ants that actually did, covered significantly shorter distances before deciding to turn around. We conclude from the observed differences in ascent and descent lengths, respectively, that the animals had access to stored information about either the elevated position of the food source, or the presence of a slope along the way. We therefore reject hypothesis A of an exclusively two-dimensional representation as the basis of the desert ants' path integration mechanism.

The percentages of flat trained animals that did not turn around immediately after stepping onto a ramp may appear large at first glance: 30 % chose to descend in the Homebound test, and ~20 % chose to ascend in the Outbound test, although the experiment's test runs marked the animals' very first exposure to a ramp. However, the short actual distances that these animals walked on ramps before turning around (Figures 2B and 5B) suggest that these tentative trips onto the ramps can probably be interpreted as explorative behavior.

### The global vector does not extend into the third dimension

The ability to perform path integration in the horizontal plane enables desert ants to walk back to their starting point on a direct, straight path, irrespective of the meanderings of the preceding outbound route [2-4]. If such a home vector extended into the third dimension (Hypothesis C), ants that were trained according to the Λ paradigm should behave like flat trained ants: eventually, the upward and downward slope cancel out in respect to the resulting vector, which is identical to that of the flat training setup. Hence, after Λ training, ants should be as reluctant as after flat training to climb down on their way home or climb up during an outbound excursion to the food source. In both cases, choosing a ramp would result in a deviation from the direction of their home-bound and food-bound vectors, respectively. Contrary to this prediction, the behavior of ants that had participated in the Λ training was strikingly different from those in the flat training, and closely resembled that of ants after ramp training (Figures 2 and 5).

The most stringent argument against a true 3-D vector (Hypothesis C) results from experiment 3, in which we tried to specifically influence the vertical component of the global vector. There was no indication whatsoever that the enforced descents had induced a (negative) vertical component of the vector – as would be postulated for a true 3-D vector. We therefore conclude that the global vector that leads desert ants back to their point of origin in the plane is essentially two-dimensional. If a walked trajectory includes a vertical component, this aspect is not accurately included into the ants' path integrator, although a correction of walking distance to ground distance takes place [16-18]. Information about ascents and descents seems to be stored as separate information about an ant's trajectory. The hypothesis of a global vector that extends to all three dimensions of space, as outlined in the introduction (Hypothesis C), is therefore rendered highly unlikely.

### Missing hills

What happens when desert ants encounter an ascent, or a hill, during repeated visits to a feeder, and are then transferred to a channel without such features? If they have stored the occurrence of such a "hill" in a general, unspecific way, its nonappearance should have an influence on the ants' search behavior. The absence of learnt visual landmarks leads to a general undershooting in the ants' homebound run [27]. Similarly, the "flat control" tests after ramp and Λ training resulted in an early onset of the ants' search for the nest entrance (Figure 4). That the ants in fact searched for the nest, and not for the "hill", can be seen by the inclusion of a training paradigm where the ramp was located at 9 m distance from the nest, instead of the usual 5.2 m distance. In the case of a search for the hill, the search distribution for the ramp at 9 m should be expected to be farther away from the position of the nest. This is not the case.

We see these results also as additional evidence that desert ants to not possess a path integrator that is operative in all three dimensions. In the case of such a mechanism employing a 3-D global vector, we would expect that at least a portion of tested animals after Λ training would welcome the opportunity to walk straight along their global vector back to the nest, and search at its correct ground distance. This is clearly not the case, with only two out of 38 animals reaching the nest position before turning around for the first time.

What remains open is the question why the search distributions of the two ramp training situations on one hand, and those after Λ training on the other, also differ from each other. Possibly, the reason for Λ training resulting in the strongest undershooting is because here an ascent and a descent were missing, or perhaps this "hill" was visually more conspicuous and was therefore a more obvious visual landmark than the descent in ramp training.

How do our findings of truncated homebound runs fit in with the results reported by Wohlgemuth *et al*. [16], where such an undershooting of walked distances did not occur? In her experiments, ants were trained to walk over a continuous series of hills and tested in a flat channel, or *vice versa*. One possible explanation is that, owing to the great discrepancy between the itinerary that was on offer during training and the subsequent test, the majority of ants exclusively relied on their global vector to find the nest. It is worth noting though that several ants in Wohlgemuth's experiments initiated their search for the nest almost immediately after being released, without running off their home vector first. In our experiment, only one ascent (in ramp training), or an ascent and a descent (in Λ training) were encountered, and the difference between the training and test situation was consequently smaller. Under these circumstances, the ants may have also tried to orientate by using these ramps as landmarks. The non-appearance of landmarks has been shown to initiate a truncation in homebound runs [27]. However, it is worth noting that even severe changes to the ants' visual environment do not prompt a reset of the path integrator, which instead is running continuously [28,29].

### Ascents and descents are not stored with their respective distances from defined points in the ants' environment

A possible way to accurately navigate between a central place (i.e. the nest) and a food source could be to extend the two-dimensional global vector to the third dimension by associating commands to climb up or down with specific values of the home vector (Hypothesis B). This would make sense especially in situations and environments in which it makes a difference where one chooses to ascend or descend – e.g. if a food source was located on a tree, and a specific trunk had to be climbed in order to reach it. In short, this means that an ant would have to remember at which exact distance from the nest (or the food source) it made a turn in the vertical dimension, and re-execute such a vertical change of direction in subsequent runs. Under this assumption, we expected in our experimental setup that after ramp training, one test ramp would be preferred, namely the ramp that was located at the same position as the ramp during the preceding training (marked by a black arrow in Figures 3 and 6). This should result in a non uniform distribution of descent/ascent frequencies with a peak at that respective ramp. The data are not in accord with this expectation for ramp trained *Cataglyphis fortis*. Neither on homebound runs nor on outbound runs did this ramp attract more descents/ascents than the other available ramps (Figures 3B, 6B). The only ramp preference of statistical relevance was for ramp No. 6 in the Homebound test, i.e. the first ramp encountered on the homebound path after ramp training. Neither the ramp training nor the Λ training (Figures 3C, 6C) give any indication for a strong preference of the test ramp located at the training distance. The inclusion of shorter descents/ascents into the analysis (Additional files 1 and 2), which can be interpreted as initial (but not final) decisions for a ramp, did not change this picture. The observation that ramp and Λ trained ants more readily walk on the first ramp they encounter in the Homebound test, but not the Outbound test, may reflect a general preference for descents. In a different experiment we conducted, ants were able to choose at one point on their way home to either ascend or descend. After all three training paradigms, the ants showed a strong preference for the downward ramp (unpublished data). One reason for this could be that the descending ramp offers unhindered vision in the forward direction during the approach of the ramp.

Hence, our results do not corroborate the hypothesis of an orientation mechanism that links commands to climb up or down to specific values of the path integrator (Hypothesis B), although such a coupling of local vectors to the overall state of the ant's path integrator has been recently demonstrated for U-turns in a flat channel [19].

### Ants do not follow the trained sequence of ascents and descents

So far, we found no evidence that ants employ a global 3-D vector when navigating in landscapes of three-dimensional formation, or that "up" and "down" commands are linked to specific states of their home vector. But how accurate is their representation of their environment with respect to the third dimension at all?

Is, for example, a hill on the ant's way recalled as the sequence of climbing up *first *and climbing down *second*? The Λ training simulates such a hill. If, in the subsequent test, the ant can choose between a straight continuation of its path and a descent, it should avoid descending on a ramp (because a preceding ascent is lacking), and continue walking horizontally – provided that it had stored the sequence of ascents and descents. In the Homebound test, however, Λ trained ants did the opposite and eagerly climbed down the offered ramps in larger numbers than after flat training (Figure 2A), with most animals covering the full length of the ramps (Figure 2B). Their behavior corresponded fully to that of ramp trained individuals. The lack of hesitation in their descents was remarkable, especially when viewed in comparison with the cautious behavior of flat trained ants, where most tested individuals soon turned around on any downward ramp that they encountered (Figure 2B). It appears that desert ants do accept any slope if they had encountered slopes before, but do not expect them to appear in the sequence as they were encountered during training. This behavior is also in accord with the hypothesis that after ramp or Λ training, a slope triggers an ant to descend, but without regard of the context within which the slope is encountered.

### Are ascents and descents local vectors themselves?

It is conceivable that stepping onto a ramp prompted the ant to follow a trained local vector leading up or down, respectively. Cues that could invoke such a local vector could be the change in proprioceptive input, caused by the changed position relative to the force of gravity, the sudden tilting of the horizon and other celestial cues, or a combination of both. Visual landmarks are known to elicit local vectors, irrespective of the status of the global vector. The Australian desert ant *Melophorus bagoti *has been shown to initiate a learned sequence of local vectors upon encountering a known landmark panorama, irrespective of the current state of its path integrator [30]. Further experiments will have to show if such local ascent/descent vectors actually exist, what properties of descents or ascents (e.g. length and slope angle) they encompass, and how they are linked to the 2-D path integrator.

## Conclusion

### Plain solutions for plane environments?

In conclusion, the abilities of desert ants to navigate within a three-dimensional environment appear to somewhat lag behind their impressive feats in two dimensions. Our findings suggest that *Cataglyphis fortis *memorizes the features of a 3-D run to a feeder only in a rather general, cursory manner. The occurrence of a sloped part of its itinerary is reflected in a general acceptance of slopes in subsequent runs. But this is neither associated with a specific point on the way between nest and food source, nor does it have to occur in the same sequence as experienced during earlier trips to the feeder.

The choices that ants made in Homebound and Outbound tests revealed that ascents and descents are neither stored with their distances from nest and feeder, nor with their correct sequence. Still, the occurrence of sloped path sections in preceding foraging trips results in more frequent and longer descents and ascents in subsequent choice experiments.

The general acceptance of slopes, given that they were encountered on earlier trips to a feeder, is reminiscent of the way how local landmarks trigger learnt changes in direction, irrespective of its congruency with the state of the path integrator [30], or the skylight compass [21].

Accepting ascents or descents on a foraging trip if they were encountered before, and rejecting them if they are new, could be a simple safety mechanism that ensures that ants do not accidentally take a "wrong turn". But such a generalized safety rule does not imply that the ants' neural representation of their environment truly possesses a property of the vertical dimension. The correction of slope distances to ground distances, coupled with the general rule not to use novel ascents and descents along a known route, may well be fully sufficient to ensure an accurate and safe orientation.

## Methods

### Experimental setup

Experiments were performed between early July and early September in the years 2004 to 2006 on desert ants (*Cataglyphis fortis*) in their natural habitat, a saltpan area at 34.52°N, 10.53°E, near Maharès, Tunisia. The ants belonged to six different nests. Each animal was tested only once.

Ants were trained to visit a feeder filled with small pieces of watermelon and biscuit crumbs. Training and experiments took place in open aluminum channels (width & height of side walls: 7 cm; see [17]). A plastic enclosure surrounded the nest entrance and guided foraging ants into the affixed training channel. Fine grey sand was glued to the channel bottom in order to increase traction. The inner side walls were painted a matt grey to prevent possibly irritating reflections from metallic surfaces. The upper end of the walls was covered with smooth adhesive tape, impeding escape attempts. Except for experiment 3, the channels provided no visually contrasting elements that could be used as landmarks or would generate optic flow cues in walking ants.

### Experiments 1 and 2

In 'reciprocal' experiments, ants were tested on their way home (experiment 1), or on their way out, towards the feeder (experiment 2).

#### Training paradigms

The ground distance from the nest to the feeder was 12 m in experiment 1, and 10.5 m in experiment 2. In both experiments, different groups of animals underwent three different training paradigms (Figure 1A–C). (i) *Flat training *took place in a straight horizontal channel. (ii) *Ramp training *utilized a channel that first led away from the nest horizontally, followed by an ascending ramp and an elevated horizontal channel. The distance of the ramp base from the nest entrance was 5.2 m in experiment 1, and 6 m in experiment 2. The length of the ramp was 150 cm in experiment 1, and 195 cm in experiment 2. The slope was 70 degrees in both cases. (iii) *"*Λ*" training *led the ants to a feeder at level ground. However, on their way they had to climb a ramp (located at the same ground distance as in ramp training), walk a short way horizontally (0.35 m), and descend a second ramp (same slope) back to ground level. The ramp lengths in (iii) corresponded to those in training (ii).

The differing dimensions in experiments 1 and 2 are owed to the possible combinations of used channel modules.

#### Test paradigms

In experiment 1, we tested the ants on their homebound run from the feeder back to the nest. After flat and Λ training, single ants were transferred to the test channel in a plastic vial filled with biscuit crumbs and released when they had taken up a morsel of food in their mandibles. In the case of ramp training, single ants carrying food were led into the adjacent test channel via a "switch" near the feeder. Neither procedure caused apparent irritations in the animals.

In the test channel, the ants encountered six "decision points" at which they could either continue to walk horizontally, or descend on a ramp (Figure 1D). All ramps used in tests were 150 cm long and had a slope of 70 degrees. The decision points were located at the following ground distances from the nest (in order of their encounter by a homebound ant): 10.5/8.9/7.3/5.7/4.2/2.6 m. "Decision points" were designed as a widening in the channel that led into two parallel channels of 7 cm width (Figure 1D, inset). One of the channels would continue horizontally, while the other led immediately to the descending ramp. On one experimental day, it would always be the channel on one side leading on horizontally at all 6 descent opportunities. The alignment was alternated between experimental days, but there were no significant differences in the results (*p *> 0.1 for all training paradigms, χ^2 ^homogeneity test). In Homebound tests, animals were tested after sufficient training, i.e. when there was a steady flow of animals that approached the feeder unhesitatingly and at high speeds. Animals were marked after testing in order to exclude them from further experiments.

In experiment 1, we also tested ants in a flat channel (length: 14 m) laid out in parallel to the training channel ('flat control'). For this control experiment, we included a further ramp training paradigm. Here, the ramp was not located at 5.2 m, but at 9 m distance from the nest (see Results section).

In experiment 2, ants were tested on their outbound run from the nest to the feeder. A switch near the nest entrance was used to guide individual ants into the test channel, laid out in parallel to the training channel. In the test channel, six ramps offering a choice to ascend or to continue on level ground, were set up at 3/4.5/6/7.5/9, and 10.5 m distance from the nest (Figure 1E). Access to the ramps was made possible by small "gateways", whose sides (each 1.75 cm wide) led up the ramp, while an opening in the center (3.5 cm wide) allowed the ants to pass through underneath the ramp (Figure 1E, inset).

In this experiment, ants were marked individually with a three dot color code of acrylic paint on their thorax and gaster. An ant had to visit the feeder at least five times prior to being tested. This ensured that an ant leaving the nest was indeed heading for the feeder. This was not necessary in Homebound tests, as a food-carrying ant will always head for home, irrespective of the number of previous visits to a food source.

### Experiment 3

#### Training paradigm

Ants were trained to walk through a horizontal channel to visit a feeder at a distance of 6 m from the nest entrance (Figure 7A). The channel was fitted out with a series of landmarks, similar to those used by Andel and Wehner [29]. These landmarks were 12 cm high, painted black on one side, and attached to the channel walls every 50 cm. By design, they were noticeable when passed on a homebound trip, while being rather inconspicuous on an outward journey. The landmarks should be associated by the animals with their homebound trip and provide an incentive to traverse the channel even when the route included a vertical diversion that was unknown to the animals, as was the case in the critical test (see below). Behind the nest and the feeder, we placed two "mock ramps" of 2 m and 1.5 m length, respectively. These ramps had no connection to the training channel, but provided a visual scenery, which matched that of the subsequent test situation. Prior to the training, ants were marked individually. Only ants that had visited the feeder at least ten times were tested, and each animal was tested only in one of the two test conditions, either the control or the critical test.

#### Test paradigms

Ants that were sufficiently trained were caught at the feeder and transferred to the test channel. Before their release, it was ensured that the ants had a biscuit crumb between their mandibles and thus were motivated to return to the nest.

In the control test, the channel consisted of a horizontal segment of 6 m length, laid out in parallel to the training channel (Figure 7A). It was fitted with the same landmarks as the training channel and ended at a test ramp (length: 2 m; slope: 70 deg). Behind the release point stood another mock ramp (1.5 m in length).

In the critical test, the release point was located in an elevated channel segment that led immediately to a descending ramp (length: 1.5 m; slope: 70 deg). At its base, the ramp connected with a horizontal channel. In 6 m ground distance from the release point, this channel ended at the base of a test ramp as in the control experiment (length: 2 m; slope: 70 deg). Both the descending ramp and the horizontal channel were again equipped with landmarks.

### Data

#### Experiment 1 and 2

We recorded the path of ants at maximum for 2 minutes, or until they had made 10 U-turns in the horizontal channel or on the ramps. The recording of the ants' behavior also ended if they had covered the full length of one of the ramps, thus clearly indicating their choice for descent or ascent. In Homebound tests, we considered an ant's run also finished when it had reached the nest-ward end of the horizontal channel. Once ants had reached this dead end, they started to search in this part of the channel for a way out, but did not walk back to the last "decision point" or beyond.

We analyzed the frequencies of animals choosing to descend or ascend a test ramp for more than 20 cm (walking distance) in Homebound and Outbound tests, respectively. If, occasionally, an individual made more than one descent or ascent, only the first one was included in the analysis. These frequencies show whether different training paradigms had an influence on the general acceptance of sloped channel segments. Furthermore, we analyzed the distance that ants walked on a ramp before the first turn in their path. Thus, we obtained not only data about a general acceptance of ramps, but also information about the stretch of a slope that an ant intended to walk after the three different training situations.

In order to examine whether any of the six available ramps was preferred over the others, we calculated the frequency of ramp choice. This was done by summing up the full descents/ascents on a ramp, as well as the sum of passes that ants had carried out at the respective "decision point". In this instance, we considered as a valid run only descents/ascents that covered the full length of the ramps. If ants turned around prior to the end of the ramp, we took this as an indication that they had ultimately decided against climbing up or down a slope at this position.

We ensured that this strict criterion for analyzing descents and ascents did not mask any results by additionally examining our data with a "softer" criterion: In this, we looked at the initial ramp choice by considering the first ramp on which the animal descended or climbed for more than 20 cm, irrespective of later turns. These results are provided in the additional files section of this article.

In the flat controls of the Homebound test, we noted the first ten turns in the ants' walked path.

#### Experiment 3

In both test situations, we recorded the length of the first ascent that an ant undertook on the test ramp. In the critical test, some ants turned around several times on the descending ramp before reaching the level segment. These turns had no influence on the length of ascent on the test ramp (*p *> 0.5; N of turning ants = 16; N of not turning ants = 21; Mann-Whitney U-test).

### Statistical analysis

#### Experiment 1 and 2

Frequencies of animals choosing to descend or ascend, as well as frequencies of chosen ramps, were analyzed using the χ^2 ^homogeneity test. If descent/ascent frequencies showed significant differences, these were localized with a pair-wise comparison using Fisher's exact test and Bonferroni correction for multiple comparisons. In the analysis of ramp choice, deviations from homogeneity were located by using the squared standardized residuals. The lengths of descents and ascents following the three training paradigms were compared for each experiment using the Kruskal-Wallis H-test. Differences between pairs of sample groups were localized using the Games-Howell post-hoc test for pair-wise comparisons. In the flat controls of the Homebound test, we used the first U-turn as an indicator for the position of an ants' search area. We compared these data between the different training paradigms using the Kruskal-Wallis H-test and Games-Howell post-hoc test.

#### Experiment 3

The length of ascents on the test ramp in the critical test (with an initial descent) and the control test (which had no descent) was compared using the Mann-Whitney U-test.

All statistical analysis was carried out using SPSS for Windows, version 12.0.1.

The experiments comply with the "Principles of animal care", publication No. 86-23, revised, 1985 of the National Institute of Health, and with the current laws of Germany and Tunisia.

## Competing interests

The authors declare that they have no competing interests.

## Authors' contributions

GG designed and conducted the majority of experiments, analysed the data, and wrote the majority of the manuscript.

RW contributed to the design of the experiments and to the writing of the manuscript.

BR designed part of the experiments, contributed to the interpretation of data and to the writing of the article.

All three authors read and approved the final version of this manuscript.

## Supplementary Material

Additional file 1 **Experiment 1: Ramp choice using a "softer" criterion for defining descents (cf**. Figure 3**)**. Tabulated are the type of preceding training and the number of decisions to either pass or descend at each of the six offered ramps, and the resulting percentage of descents. All descents of more than 20 cm were considered. Click here for file

Additional file 2 **Experiment 2: Ramp choice using a "softer" criterion for defining ascents (cf**. Figure 6**)**. Tabulated are the type of preceding training and number of decisions to either pass or ascend at each of the six offered ramps, and the resulting percentage of ascents. All ascents of more than 20 cm were considered. Click here for file
